# Successful percutaneous treatment for massive hemorrhage due to infectious pseudoaneurysm in the abdominal wall after percutaneous endoscopic gastrostomy: a case report

**DOI:** 10.1186/1756-0500-7-354

**Published:** 2014-06-10

**Authors:** Takeshi Fujita, Masahiro Tanabe, Etsushi Iida, Naofumi Matsunaga, Katsuyoshi Ito

**Affiliations:** 1 Department of Radiology, UBE INDUSTRIES, LTD. Central Hospital, 750 Nishikiwa, Ube, Yamaguchi 755-0151, Japan; 2 Department of Radiology, Yamaguchi University Graduate School of Medicine, 1-1-1 Minamikogushi, Ube, Yamaguchi 755-8505, Japan; 3 Department of Radiology, Kawasaki Medical School, 577 Matsushima, Kurashiki, Okayama 701-0192, Japan

**Keywords:** n-butyl-cyanoacrylate-lipiodol, Infectious pseudoaneurysm, Abdominal wall, Percutaneous endoscopic gastrostomy

## Abstract

**Background:**

Percutaneous endoscopic gastrostomy (PEG) is often performed for alimentation and to prevent weight loss in patients with feeding problems due to central neurologic diseases such as cerebral infarction or intracranial hemorrhage. Although infection at the skin site after PEG placement is a typical late complication of PEG, a ruptured infectious pseudoaneurysm caused massive bleeding adjacent to the tract is rare. Prompt treatment is required to avoid the hemorrhage shock, however surgical ligation is difficult to obtain the arrest of bleeding in damaged skin due to the infection.

**Case presentation:**

A 70-year-old male was bedridden due a cerebral infarction suffered 1 year previously. APEG was placed because of feeding problems, and a push-type, 20-Fr gastrostomy tube was inserted through the anterior abdominal wall. On day 16 after PEG placement, the patient had massive bleeding from the PEG site due to the rupture of infectious pseudoaneurysm and developed a decreased level of consciousness and hypotension. Treatment by percutaneous direct injection of a mixture of n-butyl-cyanoacrylate (NBCA)-lipiodol was performed and achieved good hemostasis is obtained.

**Conclusions:**

A rare case of an infectious pseudoaneurysm that developed in the abdominal wall and caused massive bleeding at a PEG placement site was described. Percutaneous injection of a mixture of n-butyl-cyanoacrylate (NBCA)-lipiodol under ultrasound guidance is an effective treatment in this case.

## Background

In iatrogenic or infectious pseudoaneurysms due to surgery or other interventions, and in superficial pseudoaneurysms due to trauma, the usefulness of surgery, embolization, ultrasound-guided manual compression, and percutaneous thrombin injection has long been reported [1-3]. The usefulness of percutaneous injection of a mixture of NBCA-lipiodol has also been described in some recent reports [4,5].

## Case presentation

A 70-year-old male was bedridden due a cerebral infarction suffered 1 year previously. A PEG was placed because of feeding problems, and a push-type, 20-Fr gastrostomy tube (Neofeed PEG kit; Top Corporation, Tokyo, Japan) was inserted through the anterior abdominal wall under electronic gastroscopy assistance (Olympus GIF 260; Olympus Corporation, Tokyo, Japan).

**Figure 1:**
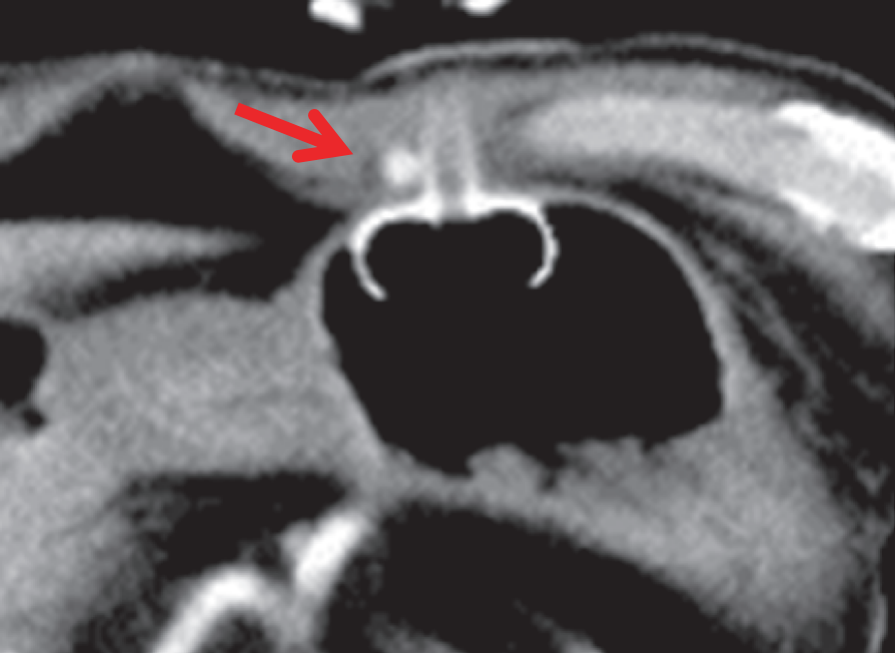
Emergency contrast-enhanced CT after hemorrhagic shock shows a well-visualized pseudoaneurysm in the right abdominal wall near the PEG tube site (arrow).

**Figure 2:**
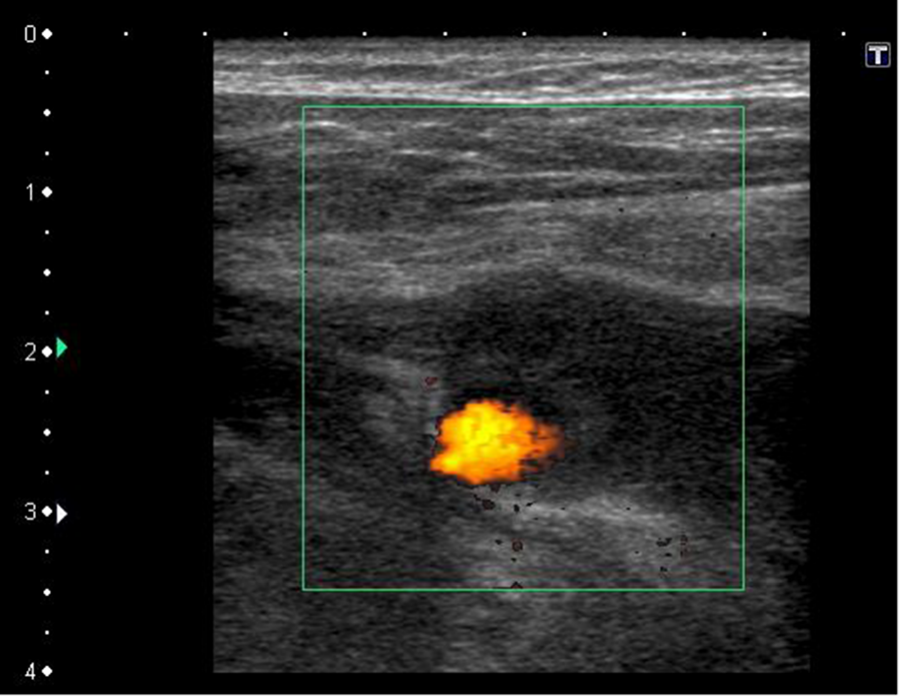
Color Doppler ultrasonography before injection therapy shows definite blood flow signal, indicating infectious pseudoaneurysm.

**Figure 3:**
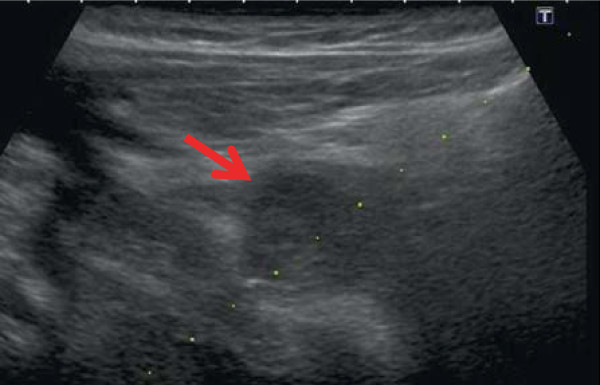
**A mixture of NBCA-lipiodol was injected into the pseudoaneurysm under echo-guidance (arrow).** The dash line indicates the echo-guided puncture line.

**Figure 4:**
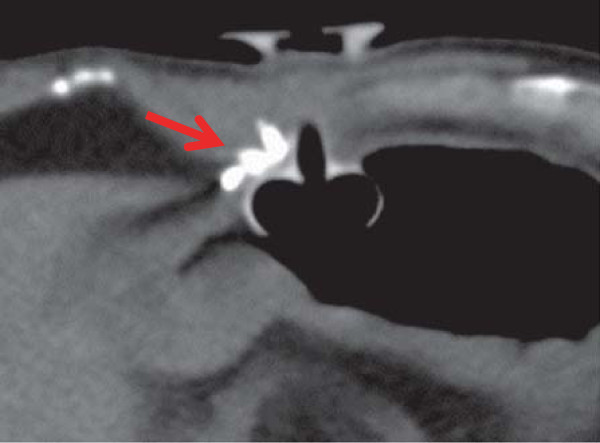
Unenhanced CT the next day after treatment shows a high-density area of lipiodol in the aneurysm (arrow).

**Figure 5:**
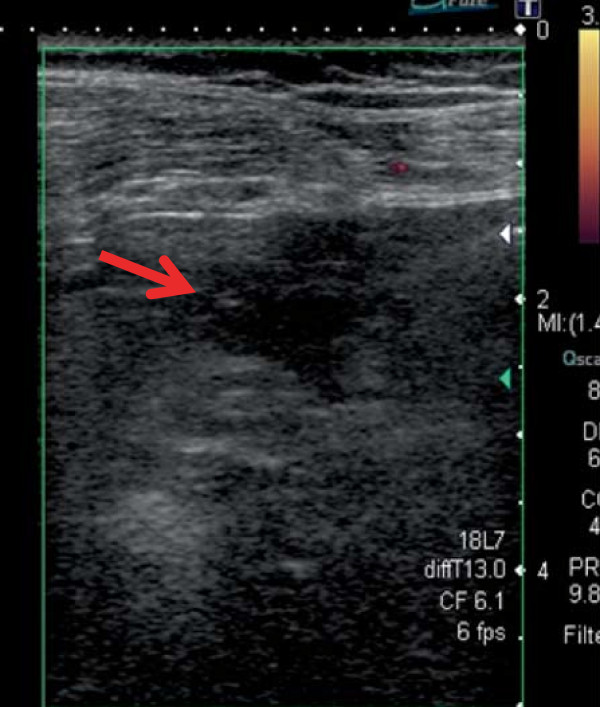
Color Doppler usltasonography one week after treatment shows loss of the blood flow signal, with complete thrombosis of the aneurysm (arrow).

CT examination after the placement of gastrostomy tube shows no abnormal findings around the gastrostomy site. An alimentation through the PEG tube was started the next day. About 13 days after PEG feeding was started, infection with erythema of skin being 60 mm in diameterat the PEG site and slight pus drainage from clearance gap between the PEG tube and skin was noted. However, the abscess formation could not be detected. Bacterial culture of the pus revealed *Pseudomonas aeruginosa*. Antibiotics to which the organism was sensitive were administered, the area was disinfected with povidone-iodine (Isodine, Meiji Seika Farma, Tokyo, Japan) and pus was carefully aspirated. Infected wound led to temporary improvement after this treatment. Thus alimentation using the PEG tube was continued. However, on day 16 after PEG placement, the patient had massive bleeding from the PEG site and developed a decreased level of consciousness and hypotension.Peripheral blood tests showed a decrease in hemoglobin from 10 mg/dL to 6 mg/dL. Hemorrhagic shock from the PEG site was diagnosed, and an emergency contrast-enhanced CT was performed. CT showed a 5-mm, nodular, enhanced mass in the right abdominal wall near the PEG tube, and an abdominal wall pseudoaneurysm due to infection was diagnosed (Figure 1). Temporary hemostasis was achieved by strong bilateral compression of the bumper of the abdominal wall and the gastric wall. However, color Doppler ultrasonography 24 h later showed blood flow in the pseudoaneurysm, so bumper compression had not achieved thrombosis (Figure 2).Therefore, the infectious pseudoaneurysm was directly punctured with a 21-G needle under ultrasound-guidance, and 0.4 mL of an NBCA-lipiodol mixture (1:1) was manually injected (Figure 3). The mixture in the infectious pseudoaneurysm hardened immediately, and color Doppler ultrasonography showed loss of the blood flow signal in the infectious pseudoaneurysm. Unenhanced CT was performed on the next day after treatment, and a high-density area in the infectious pseudoaneurysm due to lipiodol accumulation was visualized (Figure 4). One week after percutaneous injection therapy, color Doppler ultrasonography still showed that the infectious pseudoaneurysm was thrombosed without a blood flow signal (Figure 5). Use of the PEG tube was resumed 2 weeks after treatment, re-bleeding did not occur, the patient’s general condition improved, and he was discharged home. All ultrasonographic procedures were performed with an Xario scanner (Toshiba Medical Systems Corporation, Tochigi, Japan) and a 3.5-7.5 MHz transducer. This retrospective study was approved by ethical committee, and written informed consent was obtained from the patient before all procedures.

## Discussion

Complications associated with percutaneous gastrostomy tube placement are broadly divided into early complications due to the procedure, such as bleeding or bowel perforation during PEG placement, and late complications, as in the present patient, that occur sometime after PEG placement [6,7]. Most complications are infections of the skin site, and infections of the PEG tube site particularly tend to develop in patients with decreased immunity who are elderly or have poor nutritional status [6]. Infections may be mild, but abscess formation in the skin may also occur [8]. The present patient did not have abscess formation, but infection due to *Psedomonas aeruginosa* of the PEG site skin occurred, and together with this, an infectious pseudoaneurysm of the epigastric artery near the PEG tube developed.

Pseudoaneurysms in the gastric wall after PEG placement have been reported [9], but according to our literature search, an infectious pseudoaneurysm in the abdominal wall as in the present case has not been previously reported. If the infectious pseudoaneurysm ruptures, as in the present patient, bleeding can be life-threatening, and treatment such as surgery, embolization, compression, or percutaneous injection of a blood-clotting agent like thrombin is necessary [1-3]. Percutaneous injection is clearly less invasive than surgery or embolization, is convenient, and has recently become the treatment of first choice [4,5]. Various types of clotting agents are used, but when thrombin, which is the most common, is used, its potent clotting effect can severely irritate the surrounding tissue, it tends to cause allergic reactions, and embolization of peripheral vessels occasionally occurs, which can lead to surrounding tissue damage [4].

In comparison, with a mixture of NBCA-lipiodol, the NBCA immediately causes polymerization with sodium ions in the blood, so blood coagulation occurs immediately [10,11]. Thus, the blood clotting ability is potent, and irritation of the surrounding skin is less. Therefore, this is an appropriate clotting agent in cases of skin infection as in the present patient. In addition, by changing the NBCA-lipiodol mixture ratio, the clotting time can be adjusted. With a 1:1 ratio, blood polymerization and clotting are reported to occur in about 2 to 3 seconds, so selection of a 1:1 mixture ratio in the present patient led to good results [4,10,11].

## Conclusion

A rare case of an infectious pseudoaneurysm that developed in the abdominal wall and caused massive bleeding at a PEG placement site was described. Compression of the bumper was attempted, but it was unsuccessful in achieving thrombosis. The injection of a mixture of NBCA-lipiodol into the infectious pseudoaneurysm under ultrasound-guidance rapidly led to thrombosis and hemostasis. We believe that this is an effective treatment in this case.

## Consent

Written informed consent was obtained from the patient for publication of this case report and accompanying images. A copy of the written consent is available for review by the Editor-in-Chief of this journal.

## Competing interests

The authors declare that they have no competing interests.

## Authors’ contribution

TF, MT, and EI carried out clinical studies. TF prepared manuscript. NM and KI reviewed manuscript. All authors read and approved the final manuscript.
